# Supplementary material for: The origins of noise in the Zeeman splitting of spin qubits in natural-silicon devices
Source: npj Quantum Inf. 2025 Dec 9;12(1):9. doi: 10.1038/s41534-025-01150-6 (PMC12799487; doi:10.1038/s41534-025-01150-6)
Supplement: Supplementary file 1 — Supplementary Information [file 41534_2025_1150_MOESM1_ESM.pdf]

Supplementary Information for "The origins of noise in the Zeeman splitting of spin qubits in natural-silicon devices"

Juan S. Rojas-Arias,[1,*] Yohei Kojima,[2] Kenta Takeda,[2] Peter Stano,[1,3] Takashi Nakajima,[2] Jun Yoneda,[4] Akito Noiri,[2] Takashi Kobayashi,[1] Daniel Loss,[1,5] and Seigo Tarucha[1,2,†]

[1] *RIKEN, Center for Quantum Computing (RQC), Wako-shi, Saitama 351-0198, Japan*
[2] *RIKEN, Center for Emergent Matter Science (CEMS), Wako-shi, Saitama 351-0198, Japan*
[3] *Slovak Academy of Sciences, Institute of Physics, 845 11 Bratislava, Slovakia*
[4] *Tokyo Institute of Technology, Tokyo Tech Academy for Super Smart Society, Tokyo 152-8552, Japan*
[5] *Department of Physics, University of Basel, Klingelbergstrasse 82, CH-4056 Basel, Switzerland*

Supplementary Note I: Charge sensor noise spectrum

As an additional check that spectra in Fig. 1 of the main text are not dominated by charge noise, we analyze the charge sensor signal in device D1. The signal corresponds to a voltage from a reflectometry circuit which we measure at both a sensitive (on one side of a Coulomb peak of the sensor QD) and insensitive (at a valley in between two Coulomb peaks of the sensor QD) condition. In this way, we are probing purely charge noise. In order to determine the noise spectrum over a broad frequency range, we use two measurement modes. First, a fast mode, where we acquire reflectometry voltages with a rate of 1 MHz for a time span of 0.1 s. Second, a slow mode, with an acquisition rate of 1 kHz for 1 hour. The charge noise auto-PSD is obtained through the difference of the auto-PSDs at the sensitive and insensitive conditions using the method explained in Appendix G of Ref. [1]. The resulting spectrum is shown in Supplementary Figure 1. It is markedly different from spectra in Fig. 1 of the main text. Given the similarity among the spectra of qubits in the same device, if the noise were charge dominated, we would expect the sensor noise to be also similar. Since it is not, the discrepancy provides further evidence that the qubit noise is dominated by nuclear spins.

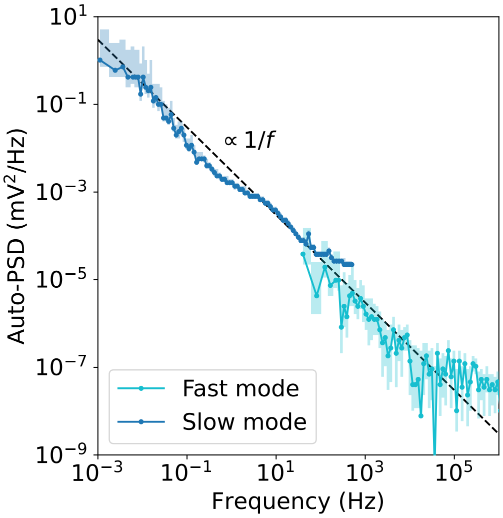

Supplementary Figure 1. **Noise auto-spectrum for the charge sensor of device D1.** The auto-PSD shown corresponds to the difference between the auto-PSDs of the charge sensor signal at a sensitive and insensitive conditions. The dashed line is a f^{-1} dependence for reference. The points correspond to the most likely estimation of the PSD with the shaded regions denoting a 90% confidence interval obtained by the methods in Ref. [1].

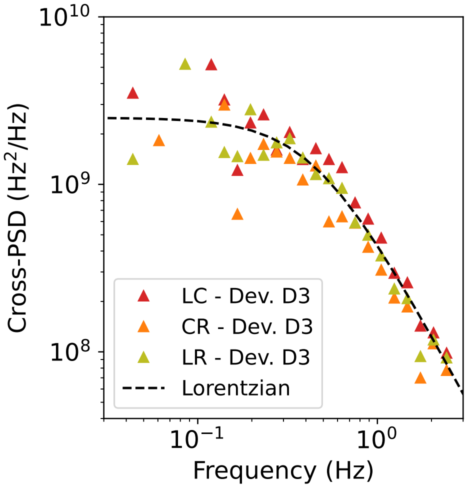

Supplementary Figure 2. **Unnormalized magnitude of the three cross-PSDs measured in device D3.** Cross-PSD of all possible qubit pairs in device D3 as colored triangles. All cross-PSDs overlap following a Lorentzian curve, as shown as a dashed line for reference.

Supplementary Note II: Unnormalized cross-PSDs of device D3

To illustrate the TLF signature in the cross-PSDs of device D3, in Supplementary Figure 2 we show the unnormalized magnitude of the cross-PSDs from device D3. The correlations follow a Lorentzian shape $\propto 1/((2\gamma)^2 + (2\pi f)^2)$, which indicates coupling to a sin-

* juan.rojasarias@riken.jp
† tarucha@riken.jp

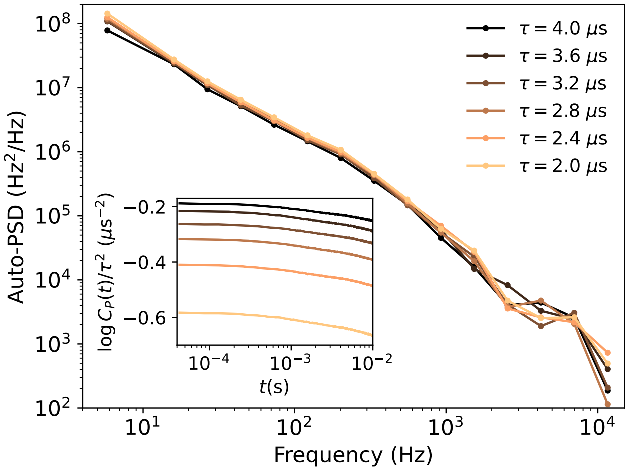

Supplementary Figure 3. **Independence of auto-PSD from single-shot correlations on evolution time.** Auto-PSDs from correlation of single-shot measurements obtained from data with different evolution times in qubit R of device D2. Inset: Logarithm of the correlator of single-shot readouts scaled by the evolution time τ.

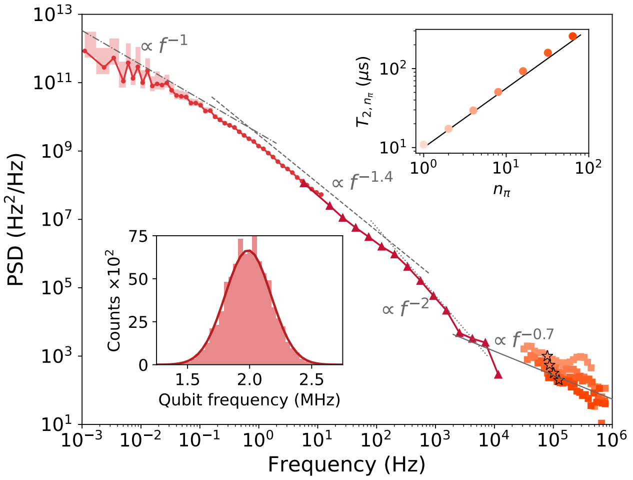

Supplementary Figure 4. **Noise auto-PSD of qubit R of device D2.** Symbols are analogous to those in Fig. 4 of the main text except for the solid gray line, which is here proportional to $f^{-0.7}$. Upper right inset: coherence time for different numbers of pulses of the CPMG sequence. The line is a $n_\pi^{0.75}$ power-law dependence drawn for reference. Lower left inset: histogram of qubit energies with a Gaussian fit as a continuous curve.

gle TLF. A fit gives its switching time $1/(2\gamma) = 0.35$ s. The cross-PSDs between first-neighbor qubits have the same amplitude as that of second neighbors. Considered together with the cross-correlation phases, some of which are negative, we conclude that the TLF responsible for these correlations is located in the array vicinity, probably near qubit C.

Supplementary Note III: Scaling of C_P with τ

When one looks at the traces $C_P(t)$ in Fig. 5 of the main text for different τ, it is not obvious that they yield the same power spectrum. The independence of the spectrum $S(f)$ on τ implied by Eq. (7) requires the functional form $C_P(t,\tau) = g(\tau)h(t)^{\tau^2}$. To demonstrate it, in the inset of Supplementary Figure 3 we plot $\log[C_P(t)]/\tau^2$ for the curves used in extracting the auto-PSD. Indeed, apart from an overall shift, the rescaled curves are very similar. Upon a Fourier transform, the shift becomes an irrelevant zero-frequency component, and we can extract the auto-PSDs from any individual curve, as shown in the main panel of Supplementary Figure 3. To benefit from the whole dataset, we use each curve and average the resulting Fourier transforms, obtaining the auto-PSDs plotted in Fig. 4 of the main text and Supplementary Figure 4.

Supplementary Note IV: Wide-range noise spectroscopy of qubit R of device D2

To show the consistency of our noise spectroscopy methods, in Supplementary Figure 4 we present the analog of Fig. 4 of the main text for qubit R of device D2. We observe a behavior similar to that of qubit L: The f^{-1} power law transforms into $f^{-1.4}$, then becomes closer to f^{-2} before becoming flatter at higher frequencies. In the insets we show the increasing coherence time with the number of dynamical decoupling pulses (upper right) as well as the Gaussian distribution of qubit frequencies in the histogram (lower left).

Supplementary Note V: Low- and high-frequency auto-PSD of qubits in device D1

In Supplementary Figure 5, we present the low- and high-frequency sections of the auto-PSDs of the qubits in device D1. The gap between these two regions highlights the importance of our newly implemented spectroscopy method based on correlation of single-shot readouts. Unlike Figure 4 of the main text and Supplementary Figure 4, where the entire frequency range is accessible, the intermediate region is not covered here. Nonetheless, the gap clearly indicates that a steeper dependence—similar to that observed in Figure 4 and Supplementary Figure 4—is required to connect the low- and high-frequency regimes for both qubits. The insets shows the scaling of the coherence time with the number of π pulses. In this device, a discrepancy in the scaling is again observed: the measured dependence follows $T_2 \propto n_\pi^{0.8}$ for qubit R and $T_2 \propto n_\pi^{0.5}$ for qubit R, whereas the auto-PSDs predicts $T_2 \propto n_\pi^{1/3}$, according to Eq. (18) in the main text.

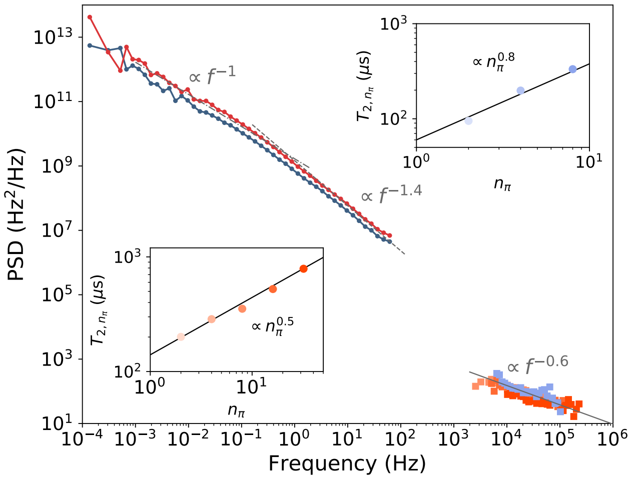

Supplementary Figure 5. **Noise auto-PSD of the qubits in device D1.** Data for qubit L is in blue, while that of qubit R is in red. Lower left inset: coherence time for different number of pulses of the CPMG sequence for qubit R. The line is a $n_\pi^{0.5}$ power-law dependence drawn for reference. Upper right inset: Analogous for qubit L, with a $n_\pi^{0.8}$ dependence for reference.

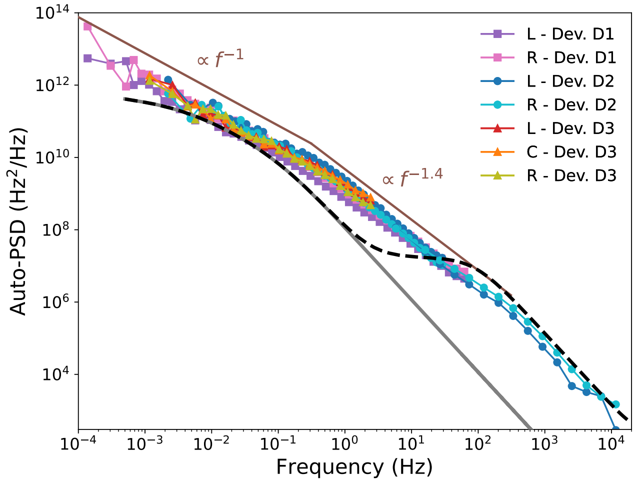

Supplementary Figure 6. **Comparison between nuclear diffusion models with and without valley coupling.** Measured auto-power spectral densities (colored symbols) are compared with theoretical models: diffusion without (gray line) and with (black dashed line) valley oscillations in the wavefunction. The latter provides a closer quantitative match to the data, particularly in the 100 Hz–10 kHz range. Both models exhibit an f^{-2} dependence at high frequencies.

Supplementary Note VI: Comparison between nuclear diffusion models with and without valley coupling

Figure 2 in the main text compares the measured auto-power spectral densities with two theoretical models: nuclear diffusion with and without valley oscillations, as described in the *Methods*. Both models reproduce the general low-frequency trend, but the inclusion of the valley term provides an improved quantitative agreement, particularly in the 10–100Hz range. Extending the comparison to a wider frequency range, as shown in Supplementary Figure 6, reveals that both models share the same high-frequency asymptotic behavior, scaling approximately as $S(f) \propto f^{-2}$. The valley-augmented model (black dashed line) more closely follows the measured spectra also in the range 100 Hz–10 kHz, whereas the model without valley coupling (gray line) retains a discrepancy of roughly two orders of magnitude across the same range.

The remaining differences between the model and the experiment are attributed to the simplifying assumptions inherent in the diffusion approach, including the treatment of the nuclear-spin bath as homogeneous. More detailed microscopic models will likely be required to capture the finer structure of the spectra.

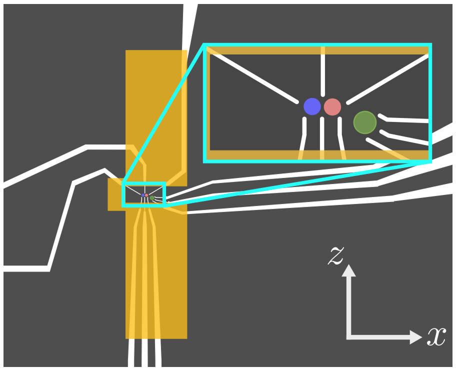

Supplementary Figure 7. **Micromagnet design.** Schematic of the cobalt micromagnet (dark yellow) used in device D1. The same design is employed in devices D2 and D3 with minor adjustments. The micromagnet is positioned such that the largest gradient lies along the qubit array and the in-plane perpendicular component ($\partial B_z/\partial z$) is minimized.

Supplementary Note VII: Simulation of magnetic field of the micromagnet

Supplementary Figure 7 shows the geometry of the cobalt micromagnet used in device D1. Devices D2 and D3 follow the same general design principle, with small variations. The micromagnets are positioned such that the dominant gradient lies along the qubit array, while the in-plane perpendicular component is minimized ($\partial B_z/\partial z = 0$).

Supplementary Figure 8 presents the simulated magnetic-field profiles and corresponding gradients produced by these micromagnets. Devices D1 and D2 share the same nominal geometry, with the micromagnet in D2 laterally shifted by 100 nm relative to that in D1. Device D3 employs a modified version of the same design with adjusted dimensions that yield a larger in-plane

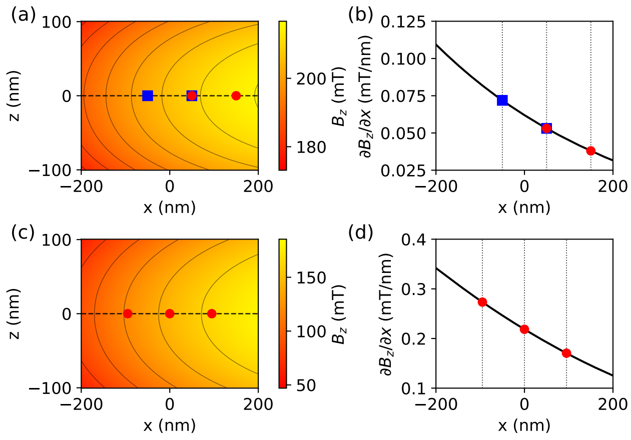

Supplementary Figure 8. **Simulation of the magnetic field profiles due to micromagnets.** (a) Simulated magnetic field profiles for devices D1 and D2. Blue squares mark the locations of the qubits in device D1, and red circles mark those in device D2. (b) Magnetic-field decoherence gradient for the same devices, with markers following the same convention as in (a). (c,d) Corresponding results for the micromagnet of device D3, with qubit locations indicated by red circles.

magnetic-field gradient. The simulations illustrate the spatial distribution of the magnetic field at the qubit locations and the relative magnitudes of the gradients that set the coupling strength between charge and spin.

The designs used here follow the concept described in Ref. [2], where the micromagnet geometry and its impact on spin-charge coupling were characterized in detail.

Supplementary Note VIII: Discrepancy in the coherence decay scaling with number of pulses

The derivation of Eqs. (16) and (18) hinges on the assumption that the noise is Gaussian. Since we observe a large discrepancy (explained in Methods), the assumption is suspect. Related to this, we note that TLFs, which are the most plausible noise source in our devices, have been long ago pointed out and extensively studied as a prototypical source of non-Gaussian noise in solid-state [3–12]. We thus examine the possibility that TLFs are the origin of the discrepancy between the prediction of Eq. (18) and the coherence time scaling observed in the data in the inset of Fig. 4 of the main text.

To this end, we set up the following procedure. We assume that the spectrum plotted in the main panel of Fig. 4 of the main text for an arbitrary fixed n_π, for example 64, is an acceptable estimate of the noise spectrum in the device. We denote it $S(f)$ and call it the 'input spectrum' in this Supplementary Note. We further assume that it is realized entirely through an ensemble of TLFs. We examine whether the observed

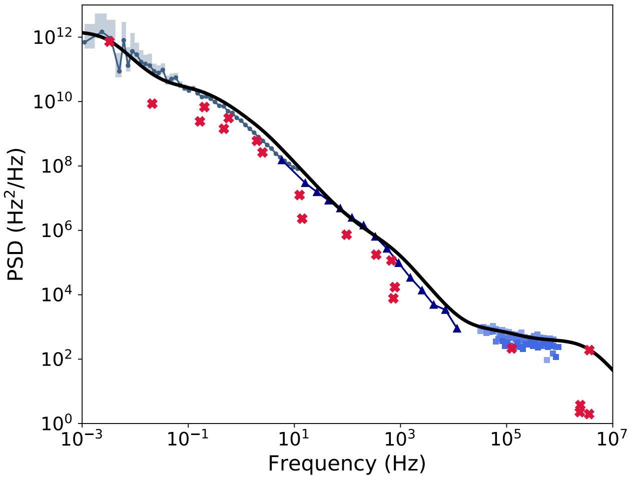

Supplementary Figure 9. **Fit of the auto-PSD with an ensemble of TLFs.** The spectral density as measured (blue symbols, data copied from the main panel of Fig. 4 of the main text) and approximated by a single sample of a random TLF ensemble. The black curve shows the ensemble spectrum $S^{\rm tlf}_{\rm tot}(f)$, defined in Eq. (S2). Each TLF is represented by a single red cross, placed at coordinates $\{f_i, S^{\rm tlf}_i(f_i)\}$ with $f_i = \gamma_i/\pi$.

discrepancy would show up in computer simulations.

A single TLF is characterized by a coupling strength v and a transition rate γ, being the average of the excitation and deexcitation rate of the TLF. A single TLF thus has a noise spectrum

$$S^{\rm tlf}(f) = \frac{1}{(2\pi)^2} v^2 \frac{2\times 2\gamma}{(2\gamma)^2 + (2\pi f)^2}, \tag{S1}$$

where we use the Fourier-transform normalization given by Eq. (8) and we intentionally leave the integer factors uncancelled, to show their origin: 2γ is the sum of the excitation and deexcitation rate of the TLF, v is the coupling strength of the TLF-qubit Hamiltonian that reads $H^{\rm tlf} = (1/2)v\sigma_z$, and the factor $(2\pi)^{-2}$ is due to $S^{\rm tlf}$ expressing the correlations of the qubit cycle frequency (and not angular frequency); see Eqs. (1) and (8).

To approximate the spectrum plotted in Fig. 4 of the main text, we sample a collection of TLFs indexed by $i = 1, 2, \ldots, M$ such that

$$S(f) \approx S^{\rm tlf}_{\rm tot}(f) \equiv \sum_i S^{\rm tlf}_i(f). \tag{S2}$$

We will describe the details of the sampling procedure elsewhere and here only give an outline: We sample the TLFs' parameters v and γ from distributions that have been established in previous literature on this topic [3, 5, 6, 9, 10]. Namely, for the coupling strength, we take the density $P(v){\rm d}v \propto v^{-(1+\mu)}{\rm d}v$, where $\mu = d/b$ with integers d and b being, respectively, the dimension of the space where TLFs are distributed randomly, and the power with which the TLF-qubit interaction falls

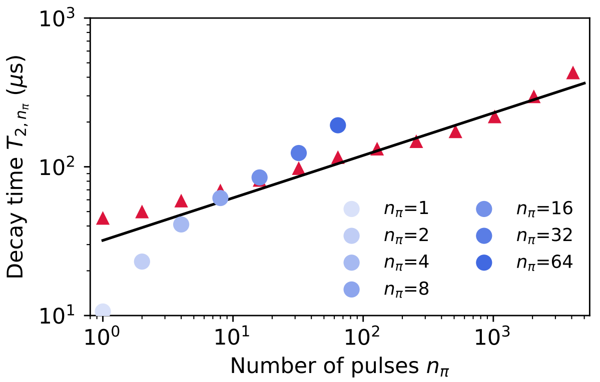

Supplementary Figure 10. **CPMG decay time as a function of the number of pulses n_π.** The triangles correspond to a TLF ensemble (the one shown in Supplementary Figure 9). The blue gradient-colored points show the decay times observed in the experiment (data copied from the inset of Fig. 4 of the main text). The solid line shows the scaling given in Eq. (18) for $\alpha = 0.4$, in correspondence with the power-law fit of the spectrum in Supplementary Figure 9 for $f > 2 \times 10^4$ Hz.

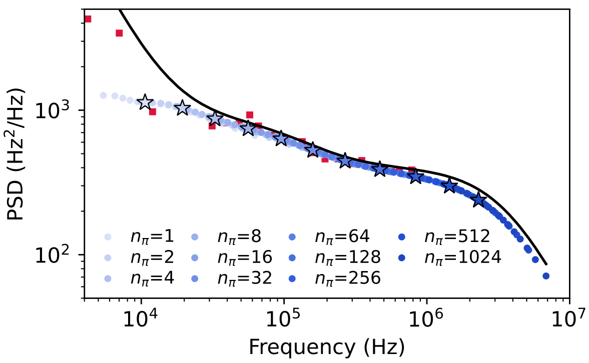

Supplementary Figure 11. **Auto-PSD reconstructed with CPMG data from an ensemble of TLFs.** The correspondence of PSD estimated from CPMG data with a fixed number of pulses n_π (varying τ) and varying n_π (with fixed τ), for noise due to an ensemble of TLFs plotted in Supplementary Figure 9. Red squares show the input spectrum $S(f)$. The black curve shows the ensemble spectrum $S^{\rm tlf}_{\rm tot}(f)$, defined in Eq. (S2). Colored points show the PSD estimates obtained using Eq. (15) for decays calculated analytically for the adopted TLF ensemble. Different colors correspond to different n_π. Stars correspond to $W = 1/e$, matching the notation of Fig. 4 of the main text.

off with their distance. Typical values are $d = 2$ or 3, and $b = 3$ or 2. For the transition rate γ, we implement power-law like slopes such as $S(f) \propto f^{-\alpha}$, that are observed in certain frequency regions in Fig. 4 of the main text, by taking $P(\gamma)\mathrm{d}\gamma \propto \gamma^{-\alpha}\mathrm{d}\gamma$.

An exemplary outcome of this procedure is in Supplementaru Figure 9 for sampling parameters that represent a sparse TLF ensemble, meaning that at most frequencies a single TLF dominates. In such a sparse regime, one can expect maximal non-Gaussian effects. Despite this possibility, we find that for noise from TLFs, one should expect an excellent correspondence between the exponents in Eqs. (16) and (18). Namely, we simulate numerically the coherence decays due to the generated TLF ensemble according to Eq. (17)-(18) in Ref. [11] (we use the forms given in Eqs. (18)-(20) in Ref. [12] which uses the TLF parameterization corresponding to Eq. (S1)).

We present the results of those simulations in two figures. Supplementary Figure 10 shows the scaling of the decay times with the number of pulses. The TLF-originated noise follows the scaling expected from the input noise spectrum, consistent with the $\alpha \approx 0.4$ slope. Replotting in the figure also the scaling observed in our experiments (points), the large discrepancy in the decay times' scaling is obvious.

An alternative visualization is given in Supplementary Figure 11. Here, we show the auto-PSD that would be estimated through Eq. (15) for signal decay W evaluated analytically for the TLF ensemble. Different from other figures where we vary n_π, here we also vary the signal decay degree, that is, the value of $W \in [0, 1]$ itself. We find excellent correspondence, which demonstrates that PSD estimates from a single decay trace or several traces are consistent. In this respect, the TLF ensemble, even if sparse, can be considered a source of Gaussian noise.

We have explored various values for the TLF-ensemble parameters, namely b, d, various cutoffs of the functions $P(v)$ and $P(\gamma)$, as well as the TLF density in space. In all cases we have observed essentially the same results. A TLF ensemble respects the correspondence between Eqs. (16) and (18). This finding is in line with the previous literature, which concluded that a large number of pulses suppresses the non-Gaussian character of TLFs [8, 10–12]. We thus conclude that the behavior that we observe in the CPMG data can not be assigned to non-Gaussian effects of TLFs.

In sum, the observed discrepancy in the coherence-time scaling is puzzling and suggests that the noise with the flat spectrum at high frequencies that we observe in two devices is neither Gaussian nor due to TLFs. It might be a novel, so far unrecognized, type of noise in these semiconductor devices. We have confirmed that qualitatively similar flat high-frequency spectrum, as well as the coherence-time scaling discrepancy, can be seen in the data from device D1 (not shown). We thank the anonymous referee for drawing our attention to this issue and for pointing out that in Ref. [13] a qualitatively similar spectrum at high frequencies (with $\alpha \approx 0.7$) was observed in a device made in purified Si (800 ppm), although in that reference, the scaling of the coherence time is consistent with the auto-PSD. We have not seen such flat noise in Ref. [14], which used a nominally almost identical device to our D1 and D2, in a different wafer with isotopically purified silicon.

Supplementary References

[1] Á. Gutiérrez-Rubio, J. S. Rojas-Arias, J. Yoneda, S. Tarucha, D. Loss, and P. Stano, Bayesian estimation of correlation functions, Physical Review Research **4**, 043166 (2022).

[2] J. Yoneda, T. Otsuka, T. Takakura, M. Pioro-Ladrière, R. Brunner, H. Lu, T. Nakajima, T. Obata, A. Noiri, C. J. Palmstrøm, A. C. Gossard, and S. Tarucha, Robust micromagnet design for fast electrical manipulations of single spins in quantum dots, Applied Physics Express **8**, 084401 (2015).

[3] Y. M. Galperin, B. L. Altshuler, and D. V. Shantsev, Low-frequency noise as a source of dephasing of a qubit, in *Fundamental Problems of Mesoscopic Physics: Interactions and Decoherence*, edited by I. V. Lerner, B. L. Altshuler, and Y. Gefen (Springer Netherlands, Dordrecht, 2004) pp. 141–165.

[4] G. Ithier, E. Collin, P. Joyez, P. J. Meeson, D. Vion, D. Esteve, F. Chiarello, A. Shnirman, Y. Makhlin, J. Schriefl, and G. Schön, Decoherence in a superconducting quantum bit circuit, Physical Review B **72**, 134519 (2005).

[5] Y. M. Galperin, B. L. Altshuler, J. Bergli, and D. V. Shantsev, Non-Gaussian Low-Frequency Noise as a Source of Qubit Decoherence, Physical Review Letters **96**, 097009 (2006).

[6] J. Schriefl, Y. Makhlin, A. Shnirman, and G. Schön, Decoherence from ensembles of two-level fluctuators, New Journal of Physics **8**, 1 (2006).

[7] J. Bergli and L. Faoro, Exact solution for the dynamical decoupling of a qubit with telegraph noise, Physical Review B **75**, 054515 (2007).

[8] Ł. Cywiński, R. M. Lutchyn, C. P. Nave, and S. Das Sarma, How to enhance dephasing time in superconducting qubits, Physical Review B **77**, 174509 (2008).

[9] J. Bergli, Y. M. Galperin, and B. L. Altshuler, Decoherence in qubits due to low-frequency noise, New Journal of Physics **11**, 025002 (2009).

[10] E. Paladino, Y. M. Galperin, G. Falci, and B. L. Altshuler, 1/f noise: Implications for solid-state quantum information, Reviews of Modern Physics **86**, 361 (2014).

[11] G. Ramon, Non-Gaussian signatures and collective effects in charge noise affecting a dynamically decoupled qubit, Physical Review B **92**, 155422 (2015).

[12] M. Mehmandoost and V. V. Dobrovitski, Decoherence induced by a sparse bath of two-level fluctuators: Peculiar features of $1/f$ noise in high-quality qubits (2024), arXiv:2404.18659 [cond-mat, physics:quant-ph].

[13] R. M. Jock, N. T. Jacobson, M. Rudolph, D. R. Ward, M. S. Carroll, and D. R. Luhman, A silicon singlet–triplet qubit driven by spin-valley coupling, Nature Communications **13**, 641 (2022).

[14] J. Yoneda, K. Takeda, T. Otsuka, T. Nakajima, M. R. Delbecq, G. Allison, T. Honda, T. Kodera, S. Oda, Y. Hoshi, N. Usami, K. M. Itoh, and S. Tarucha, A quantum-dot spin qubit with coherence limited by charge noise and fidelity higher than 99.9%, Nature Nanotechnology **13**, 102 (2018).
